# Factor H binding protein (fHbp)-mediated differential complement resistance of a serogroup C *Neisseria meningitidis* isolate from cerebrospinal fluid of a patient with invasive meningococcal disease

**DOI:** 10.1099/acmi.0.000255

**Published:** 2021-09-09

**Authors:** Alessandra Facchetti, Jun X. Wheeler, Caroline Vipond, Gail Whiting, Hayley Lavender, Ian M. Feavers, Martin C. J. Maiden, Sunil Maharjan

**Affiliations:** ^1^​ Division of Bacteriology, National Institute for Biological Standards and Control (NIBSC), Blanche Lane, South Mimms, Potters Bar, Hertfordshire, EN6 3QG, UK; ^2^​ Division of Analytical Biological Sciences, National Institute for Biological Standards and Control (NIBSC), Blanche Lane, South Mimms, Potters Bar, Hertfordshire, EN6 3QG, UK; ^3^​ Sir William Dunn School of Pathology, University of Oxford, South Parks Road, Oxford, OX1 3RE, UK; ^4^​ Department of Zoology, Peter Medawar Building, University of Oxford, South Parks Road, Oxford, OX1 3SY, UK

**Keywords:** Bexsero, factor H binding protein, fHbp, MenC, *Neisseria meningitidis sero* group C, proteomic, TMT-MS, university outbreak

## Abstract

During an outbreak of invasive meningococcal disease (IMD) at the University of Southampton, UK, in 1997, two *Neisseria meningitidis* serogroup C isolates were retrieved from a student (‘Case’), who died of IMD, and a close contact (‘Carrier’) who, after mouth-to-mouth resuscitation on the deceased, did not contract the disease. Genomic comparison of the isolates demonstrated extensive nucleotide sequence identity, with differences identified in eight genes. Here, comparative proteomics was used to measure differential protein expression between the isolates and investigate whether the differences contributed to the clinical outcomes. A total of six proteins were differentially expressed: four proteins (methylcitrate synthase, PrpC; hypothetical integral membrane protein, Imp; fructose-1,6-bisphosphate aldolase, Fba; aldehyde dehydrogenase A, AldA) were upregulated in the Case isolate, while one protein (Type IV pilus-associated protein, PilC2) was downregulated. Peptides for factor H binding protein (fHbp), a major virulence factor and antigenic protein, were only detected in the Case, with a single base deletion (ΔT366) in the Carrier fHbp causing lack of its expression. Expression of fHbp resulted in an increased resistance of the Case isolate to complement-mediated killing in serum. Complementation of fHbp expression in the Carrier increased its serum resistance by approximately 8-fold. Moreover, a higher serum bactericidal antibody titre was seen for the Case isolate when using sera from mice immunized with Bexsero (GlaxoSmithKline), a vaccine containing fHbp as an antigenic component. This study highlights the role of fHbp in the differential complement resistance of the Case and the Carrier isolates. Expression of fHbp in the Case resulted in its increased survival in serum, possibly leading to active proliferation of the bacteria in blood and death of the student through IMD. Moreover, enhanced killing of the Case isolate by sera raised against an fHbp-containing vaccine, Bexsero, underlines the role and importance of fHbp in infection and immunity.

## Introduction

*Neisseria meningitidis *, the meningococcus, is a human-specific Gram-negative bacterium, which resides asymptomatically in the oropharynx of ~10–35 % of the population [1, 2, 3]. On occasion, the bacterium causes invasive meningococcal disease (IMD), which is clinically manifested as meningitis and/or septicaemia [4]. Six (serogroups A, B, C, W, Y, X) of the 12 meningococcal capsular serogroups are responsible for the majority of cases worldwide, with infants, teenagers and university students living in halls of residence among those exhibiting the highest risks of developing IMD [5, 6].

In the 1990s, serogroup C (MenC) meningococcal isolates belonging to the hypervirulent clonal complex ST-11/ET-37 (cc11) were responsible for the majority of IMD cases in Europe [7]. During this period, the UK experienced an increase in the number of serogroup C (MenC) cases caused by this clonal complex (c:CC11), mostly occurring amongst students living in university halls of residence in England and Wales [8, 9, 10]. The introduction of a MenC conjugate vaccination programme in the UK in 1999 resulted in the reduction of both MenC cases and carriage rates [11, 12].

In 1997, a meningococcal outbreak at the University of Southampton (UK) resulted in the death of three students over a period of 19 days [13]. Ten c:CC11 isolates were retrieved and characterized using multilocus sequence typing (MLST), serological (PorA and PorB) and PFGE techniques [14]. Two of these isolates showed identical MLST, serological and PFGE profiles, with no similarities to the remaining isolates, suggesting they were part of a smaller outbreak [15]. While one isolate, referred to as ‘Case’, was recovered from the cerebrospinal fluid (CSF) of a student who died of IMD, the other, ‘Carrier’, was isolated from the oropharynx of a person who performed mouth-to-mouth resuscitation on the student who died but did not contract the disease [14]. Isolates were later interrogated using whole genome sequencing (WGS) [15], which confirmed their high degree of sequence identity at the level of WGS and identified differences in only eight genes, with 163 nucleotide changes identified including single nucleotide polymorphisms (SNPs) and deletions.

In the present study, we employed comparative proteomics to further characterize the Case and Carrier isolates from the 1997 University of Southampton outbreak to identify proteins differentially expressed that could have affected the ability of the Case isolate to cause disease and death of a student. Proteome comparison techniques have been previously applied to *N. meningitidis* isolates to measure protein expression under different culture conditions as well as to investigate protein links and functions [16; 17; 18]. We then investigated differences in the phenotypes of the isolates by measuring their ability to survive in human serum and investigated underlying genetic mechanisms to better understand how the observed evolutionary changes may have occurred.

## Methods

### Bacteria strains and growth conditions

The following *N. meningitidis* isolates were used in this study: MenC ‘Case’ (id 41784 on PubMLST *Neisseria* database https://pubmlst.org/neisseria/ [19], isolate NIBSC_2839), MenC ‘Carrier’ (id 41785, NIBSC_2838), *N. meningitidis* serogroup B (MenB) H44/76 (id 237, NIBSC_2851) and MenC PubMLST id 644 (NIBSC_2759).

Bacteria from −80 °C stocks were grown on Mueller-Hinton blood agar (MHBA) plates, where Mueller-Hinton (MH) agar (Oxoid, Thermo Fisher Scientific) was supplemented with 5 % horse blood (TCS Biosciences), and incubated overnight at 37 °C in the presence of 5 % CO_2_. For liquid cultures, bacterial colonies from overnight incubation on MHBA plates were inoculated into ~5 ml MH broth (Oxoid, Thermo Fisher Scientific) to obtain a starting OD_600nm_ of ~0.2 and grown at 37 °C overnight with 180 r.p.m. shaking. OD_600nm_ was measured to identify bacterial growth using 1.6 ml disposable polystyrene cuvettes with 10 mm path length (Fisherbrand) in a densitometer (Biowave Cell Density Meter C08000).

### PubMLST and alignments

Sequences for genes and proteins investigated in this study were extracted from the annotated whole genome available in the PubMLST *Neisseria* database aligned using BioEdit (v.7.0.5).

### Tandem mass tag MS

Case and Carrier isolates were grown on MHBA plates overnight as described. A few colonies were transferred into a Chemically Defined Medium (Thermo Fisher Scientific and Sigma-Aldrich) broth [20] to obtain an OD_600nm_ of ~0.2 and isolates were grown in a shaking incubator at 37 °C for 5–6 h until an OD_600nm_ of ~0.6 (~1×10^6^ c.f.u. ml^−1^) was reached. Following heat inactivation for 30 min at 56 °C, 1 ml of each bacterial suspension was centrifuged, and the pellet washed twice in 0.85 % NaCl (in house). Bacteria were then lysed (200 mM TEAB from Thermo Fisher Scientific; 0.5 % SDS at pH 8.0, DNase, RNase and protease inhibitors from Sigma-Aldrich) and protein concentration was measured using a BCA assay (Thermo Fisher Scientific) according to the manufacturer’s instructions.

The tandem mass tag (TMT) labelling procedure followed the manufacturer’s recommendations (Thermo Fisher Scientific). In brief, protein lysates from equal amounts of three biological replicates of Case and Carrier isolates were reduced with tris(2-carboxyethyl) phosphine and alkylated with iodoacetic acid before an overnight acetone precipitation. Protein pellets were digested overnight at 37 °C in 200 mM TEAB solution containing 2.5 µg trypsin (Promega) with the resulting peptides labelled with different isobaric tags (TMTs 126–128). Labelled peptides were mixed and injected onto an XBridge C18 column (5 µm, 4.6 mm inner diameter and 25 cm long; Waters) for the first dimension high pH reversed-phase HPLC separation under a linear gradient consisting of mobile phase A (10 mM ammonium formate, pH 10.0) and up to 70 % B (90 % acetonitrile in mobile phase A) for 2 h at a flow rate of 0.5 ml min^−1^, using a Jasco system consisting of an autosampler, semi-micro HPLC pumps and UV detector. Eluted fractions were collected and concentrated into 12 tubes and vacuum dried.

Nano-LC and MS/MS were performed using a U3000 direct nano system coupled with a nano-electrospray and LTQ-Orbitrap Discovery mass spectrometer (Thermo Fisher Scientific). The 18 HPLC fractions containing the mixture of TMT6ple- labelled peptides were resuspended in 0.1 % formic acid and each was separated on a PepMap C18 reversed-phase nano column (3 µm, 10 nm, 50 cm length; Thermo Fisher Scientific) under a column flow rate of 0.3 µl min^−1^ using a linear gradient of 5–25 % for 180 min, 25–32 % for 20 min and 32–90 % for 10 min of 95 % acetonitrile and 0.1 % formic acid. MS scanning and MS/MS fragmentation were carried out in an Orbitrap and LTQ respectively using two cycles of the top three data-dependent acquisitions with dynamic exclusion mode enabled and total cycle time at ˜30 ms. The first cycle used collision-induced dissociation (CID) fragmentation generating spectra for peptide sequencing, and the second cycle used high-energy CID (HCD) generating spectra both for peptide sequencing and for relative quantitation via report ions.

Mass spectra processing, database searching and quantification were performed using Thermo Proteome Discoverer 1.4 with built-in Sequest against the UniProt *N. meningitidis* serogroup C FASTA database (release 2014.04.03). Spectra from the 18 fractions were added together as one sample during searching. Initial mass tolerances by MS were set to 10 p.p.m. Trypsin was used as a cleavage enzyme and a maximum of two missed cleavage sites were allowed. Methionine oxidation was set as a dynamic modification whereas carboxymethylation on cysteine, TMT6plex labels on N-terminal amino acid and lysine side chain were set as static modifications. Peptides at rank 1 with high confidence are considered to be unambiguously sequenced. Quantification was based on the relative abundances of TMT tag as the reporter ions for each peptide in the HCD spectra with all TMT channels present. Ratios were calculated from relative abundances of each labelled peptide in the sample based on reporter ion intensities and for every protein identified, and each was assigned a series of quantification ratio relative to each group.

### Isolation of total RNA and qRT-PCR

Case and Carrier isolates were grown in MH broth to an OD_600nm_ of ~0.6 and heat-inactivated as described. Two volumes of RNAprotect (Qiagen) were mixed with one volume of bacterial culture to protect RNA from enzymatic degradation, followed by incubation at room temperature for 5 min. Cells were harvested by centrifugation at 9000 ***g*** for 15 min and total RNA was extracted from the cell pellets using a RNeasy Mini kit (Qiagen). DNA was removed by on-column RNase-free DNase digestion (Qiagen). RNA was then eluted using 50 µl RNase-free water (Qiagen) and quantified via Qubit fluorometric quantification (Thermo Fisher Scientific).

Custom primers and probes for quantitative reverse transcriptase PCR (qRT-PCR) (Table S1) were designed using Thermo Fisher Scientific primers and probes online design software (https://www.thermofisher.com/uk/en/home/life-science/oligonucleotides-primers-probes-genes.html). qRT-PCR was performed in MicroAmp Fast Optical 96-Well Reaction Plates sealed with MicroAmp Optical Adhesive Film (Applied Biosystems). Twenty-five microlitres of reaction was set up using 1× TaqMan RT Enzyme Mix, 1× TaqMan RT-PCR Mix (both from the TaqMan RNA-to-CT 1-Step kit; Applied Biosystems), 500 nM of each target and control primers and 250 nM of target probe. Two microlitres (approx. 50 ng) of total meningococcal RNA for each isolate was added to each separate reaction. Expressions of target genes were normalized using the housekeeping gene glucose dehydrogenase (*gdh*) and then further normalized against the Carrier isolate, which was set as the calibrator strain.

### SDS-PAGE and western blot

Whole cells were obtained from 2 ml overnight cultures in MH broth as described previously and centrifuged for 5 min at 14 000 ***g***. Pellets were then resuspended in 0.5 ml of 1× PBS+0.05 % SDS (in house and Sigma-Aldrich). To reduce viscosity due to DNA, 10 µl DNAse (Qiagen) was added and tubes were placed on a shaker, at room temperature, for 1 h. The suspensions were then centrifuged at 14 000 ***g*** for 15 min and supernatants were harvested for SDS-PAGE and western blotting. DNA and protein concentrations were measured using Qubit fluorometric quantification (Thermo Fisher Scientific).

Approximately 7 µg of total protein for each sample was loaded onto an 8–18% western blot (WB) gel card and run on an electrophoresis unit (both Amersham WB system; GE Healthcare). Proteins were transferred from the gel to a PVDF membrane card (Amersham WB; GE Healthcare). The membrane was blocked in 5 % BSA (Sigma-Aldrich), followed by incubation with mAb JAR5 (NIBSC 13/216, used at 1:1000 dilution), raised against factor H binding protein (fHbp), or polyclonal mouse sera raised against Outer Membrane Protein Class 4, RmpM (in house, NIBSC, 1:1000 dilution), as an internal loading control. Bound JAR5 and RmpM antibodies were detected using goat anti-mouse Cy5 secondary antibody (Thermo Fisher Scientific, 1:2500 dilution) and a fluorescence scanner (Amersham WB system; GE Healthcare). MenB H44/76 isolate expressing fHbp variant 1 (V1) and MenC isolate id 644 expressing fHbp variant 2 (V2) were used respectively as positive and negative controls for fHbp V1 in western blots.

### Modification of the Carrier isolate for expression of IPTG-inducible fHbp

The Carrier isolate was modified to express fHbp variant 1 (V1, from MC58), the same fHbp variant expressed by the Case isolate. This protein was expressed at an unlinked locus in the Carrier genome using pGCC4 erythromycin-resistant (erm^R^) plasmid [21, 22, 23], kindly provided by Professor C. M. Tang (Sir William Dunn School of Pathology, University of Oxford). The pGCC4 plasmid construct expressing fHbp, referred to as ‘pGCC4-fHbp’ (Fig. S1, available in the online version of this article), contained an IPTG-inducible copy of fHbp V1 cloned in the intergenic region between *lctP* and *aspC* loci of an pGCC4 ‘empty’ plasmid, referred to as ‘pGCC4’ (Fig. S1), at *Pac*I and *Fse*I restriction sites.

Both plasmids were linearized using *Cla*I (New England Biolabs) and gel-purified, and fragments (approx. 1 µg) were added separately to 2 ml of Carrier bacterial culture in MH broth containing 1 mM MgCl_2_ (Sigma-Aldrich) to allow transformation and homologous recombination. The mixtures were incubated at 37 °C for 4 h with 180 r.p.m. shaking. Fifty microlitres of transformation mixes was then plated on MHBA plates containing erythromycin (5 µg ml^−1^) and incubated overnight at 37 °C, 5 % CO_2_. The erythromycin-resistant transformants, referred to as ‘erm^R^-Carrier’ (Carrier isolate transformed with linearized ‘empty’ pGCC4) and ‘erm^R^-fHbp-Carrier’ (Carrier transformed with linearized ‘pGCC4-fHbp’) were screened by PCR (2× Phusion Master Mix; Thermo Fisher Scientific) for the absence or presence of fHbp respectively, using the primers listed in Table S1. fHbp expression in the erm^R^-fHbp-Carrier isolate was later achieved by incubation of broth cultures overnight with 1 mM IPTG (Thermo Fisher Scientific) at 37 °C, 5 % CO_2_.

### Whole-cell ELISA

For preparation of whole cell lysates, colonies from overnight cultures on MHBA plates of the Carrier, Case, erm^R^-Carrier and erm^R^-fHbp-Carrier were inoculated into 10 ml MH broth and adjusted to an OD_600nm_ of ~0.2. Isolates were grown at 37 °C, 5 % CO_2_, 180 r.p.m. shaking until an OD_600nm_ of ~0.6 was reached, with the addition of an overnight growth step in MH broth supplemented with 1 mM IPTG (Thermo Fisher Scientific) to allow fHbp expression in the erm^R^-fHbp-Carrier. Cells (2 ml) were then centrifuged for 5 min at 14 000 ***g***. Pellets were resuspended in 1 ml of 1× PBS (in house) and heat-inactivated as previously described. Whole cell lysates were then diluted 1 in 10 in 1× PBS (in house) to coat ELISA plates (Nunc flat bottom MicroWell 96-well microplates; Thermo Fisher Scientific), which were dried in a fan-assisted incubator at 37 °C overnight. The following day, plates were blocked with 1× PBS (in house), 5 % fetal calf serum (FCS; Biosera) at room temperature for 1 h. Anti-fHbp mAb JAR5 (NIBSC 13/216, used at 1:2000 dilution) or anti-meningococcal serogroup A mAb (NIBSC 95/674, 1:2000 dilution), as a negative control, were added for 1 h, followed by 1 h of incubation with goat anti-mouse IgG–HRP (Sigma-Aldrich, 1:20 000 dilution). TMBlue solution (Leinco Technologies) was used for developing the reaction, which was stopped after 15 min with 1 M sulphuric acid (in house). OD_450nm_ values were then measured using a plate reader (SoftMax Pro v6; Molecular Devices).

### Serum sensitivity assay

Serum sensitivity assays were performed as described previously [24] with slight modifications. After overnight growth of the Case, Carrier, erm^R^-Carrier and erm^R^-fHbp-Carrier strains in MH broth (with the addition of an overnight growth step in MH broth supplemented with 1 mM IPTG for erm^R^-fHbp-Carrier), suspensions were adjusted to an OD_600nm_ of ~ 0.2 and then diluted 1:2500 in 1× PBS (in house). Ten microlitres of each diluted culture was incubated with 40 µl of 20 % normal human serum (NHS; CDC1992, NIBSC 99/706; diluted in 1× PBS) at 37 °C, 5 % CO_2_ for 1 h, with shaking at 180 r.p.m. Following incubation, 10 µl from each well was plated onto square MHBA plates (Thermo Fisher Scientific) by tilting and plates were incubated overnight at 37 °C, 5 % CO_2_. Survival was calculated as the percentage of cells grown after incubation with 20 % NHS vs. 20 % heat-inactivated (HI) NHS, which was used as a growth control, where serum was heat inactivated at 56 °C for 30 min.

### Immunization of mice

Groups of eight female CD1 mice (18–22 g in weight) were immunized (Home Office Project Licence: PPL PE12BE3A7) with Bexsero (GlaxoSmithKline), a vaccine consisting of three recombinant proteins, fHbp, Neisseria heparin binding antigen (NHBA) and Neisseria adhesin A (NadA) alongside outer membrane vesicles (OMVs) from strain NZ98/254 [25]. Mice were immunized with a 1:160 dilution of Bexsero standard human dose (for a total of 0.313 µg of each recombinant protein and 0.156 µg of OMVs) diluted in 0.85 % sterile saline (in house). Immunization was achieved by intraperitoneal injection at day 0, followed by a booster at day 21 with the same vaccine lot and dilution. Blood (~1–1.5 ml) was collected on day 35 by terminal bleed. Sera were then isolated by centrifugation at 13 000 ***g*** for 10 min and stored at −20 °C. Sera from mice immunized with six different lots of Bexsero were pooled for testing in a Serum Bactericidal Antibody assay.

Sera from mice immunized with NadA or NHBA antigens, kindly provided by Dr B. Bolgiano (NIBSC), were used 1:2000 in a whole-cell ELISA, while sera from mice immunized with 0.85 % saline (in house), kindly provided by Dr H. Chan, were used as a negative control.

### Serum bactericidal antibody assay

Serum bactericidal activity of sera from mice immunized with Bexsero was evaluated against the MenC Carrier and Case isolates, as described elsewhere with some modifications [26]. A few colonies of the Carrier and Case isolates from overnight cultures on MHBA plates were transferred onto fresh MHBA plates and grown at 37 °C, 5 % CO_2_ for 4 h. Bacteria were resuspended in bactericidal buffer (Gey’s balanced salt solution +0.5 % BSA; both Sigma-Aldrich) to an OD_600nm_ of ~0.2. The suspensions were diluted 1:2500 to obtain a cell concentration of approx. 5×10^4^ cells ml^−1^. Equal volumes (10 µl) of the diluted cells and baby rabbit complement (Pel-Freeze Biologicals) were added to 20 µl of Bexsero pooled sera serially diluted two-fold in bactericidal buffer in a 96-well polypropylene U-bottom microtitre plate (Greiner Bio-One), which was then incubated at 37 °C, 5 % CO_2_ for 1 h. Following incubation, 10 µl from each well was removed and allowed to flow on square MHBA plates (Thermo Fisher Scientific) by tilting, before plates were incubated overnight at 37 °C, 5 % CO_2_. Colony-forming units in different Bexsero pooled sera dilutions were counted and compared to those observed in the control wells where heat-inactivated Bexsero pooled sera were used. The reciprocal of the serum dilution that resulted in 50 % killing relative to the controls was assigned as the SBA titre for each isolate.

### Statistical analysis

Standard deviation and statistical significance were evaluated using Minitab (LLC) and Prism v.7 (GraphPad). Significance was assigned using *P* values: *P<0.05, **P<0.01, ***P<0.001, ****P<0.0001.

## Results

### Identification of proteins differentially expressed between the Case and Carrier isolates

Whole proteome comparison performed on three independent protein lysates for the Case and Carrier isolates using TMT-MS LC-MS/MS identified 848 proteins. Arbitrary cut-off values of protein fold changes of <0.8 and > 1.2 were applied and six proteins were identified as differentially expressed between the Case and Carrier isolates (Table 1). Peptides for fHbp were detected in the Case, but not in the Carrier isolate. In addition to fHbp, four proteins were upregulated in the Case isolate: methylcitrate synthase (PrpC), hypothetical integral membrane protein (Imp), fructose-1,6-bisphosphate aldolase (Fba) and aldehyde dehydrogenase A (AldA). Protein fold changes for these proteins ranged between 1.5 and 2.99, as shown in Table 1. One protein, Type IV pilus-associated protein (PilC2), was downregulated in the Case compared to the Carrier isolate, with a protein fold change of 0.05.

**Table 1.** Comparative proteomics using TMT-MS identified six differentially expressed proteins between the Case and Carrier isolates. Relative quantification (RQ) values of gene expression obtained via qRT-PCR are also shown for *prpC*, *imp*, *fba* and *aldA* genes. Values have been normalized using both *gdh* and the Carrier isolate as calibrators.

Locus	Protein	Protein fold changes (Case/Carrier)	RQ of relative gene expression (Case vs. Carrier)	Functions	Status
**NEIS0349**	Factor H binding protein (**fHbp**)	32.46	Not measured	Virulence factor; inhibitor of complement-mediated killing in serum	Expressed by the Case isolate only
**NEIS1732**	Methylcitrate synthase (**PrpC**)	2.99	29.28	Transferase activity	Upregulated in the Case isolate
**NEIS1147**	Hypothetical integral membrane protein (**Imp**)	2.21	47.11	Hypothetical membrane protein	
**NEIS0350**	Fructose-1,6-bisphosphate aldolase (**Fba**)	1.81	15.94	Metabolism; binding to human plasminogen; additional ‘moonlight’ functions	
**NEIS1942**	Aldehyde dehydrogenase A (**AldA**)	1.50	20.91	Oxidoreductase activity	
**NEIS0033**	Type IV pilus-associated protein (**PilC2**)	0.05	Not measured	Adhesion	Downregulated in the Case isolate

The upregulation of *prpC, imp, fba* and *ald*A in the Case were confirmed by qRT-PCR, which revealed increased gene transcription, with relative quantification (RQ) values ranging between 15.94 and 47.11 (Table 1).

### fHbp expression in the isolates

Analysis of *fHbp* nucleotide sequences in the isolates revealed a nucleotide base deletion (ΔT366) in the gene of the Carrier isolate. This deletion introduces a stop codon at nucleotide 400, amino acid position 134, resulting in the absence of fHbp expression in the Carrier isolate. Lack of fHbp expression in other *N. meningitidis* isolates containing this deletion (‘fHbp allele 669') was also previously reported [27].

**Fig. 1.**
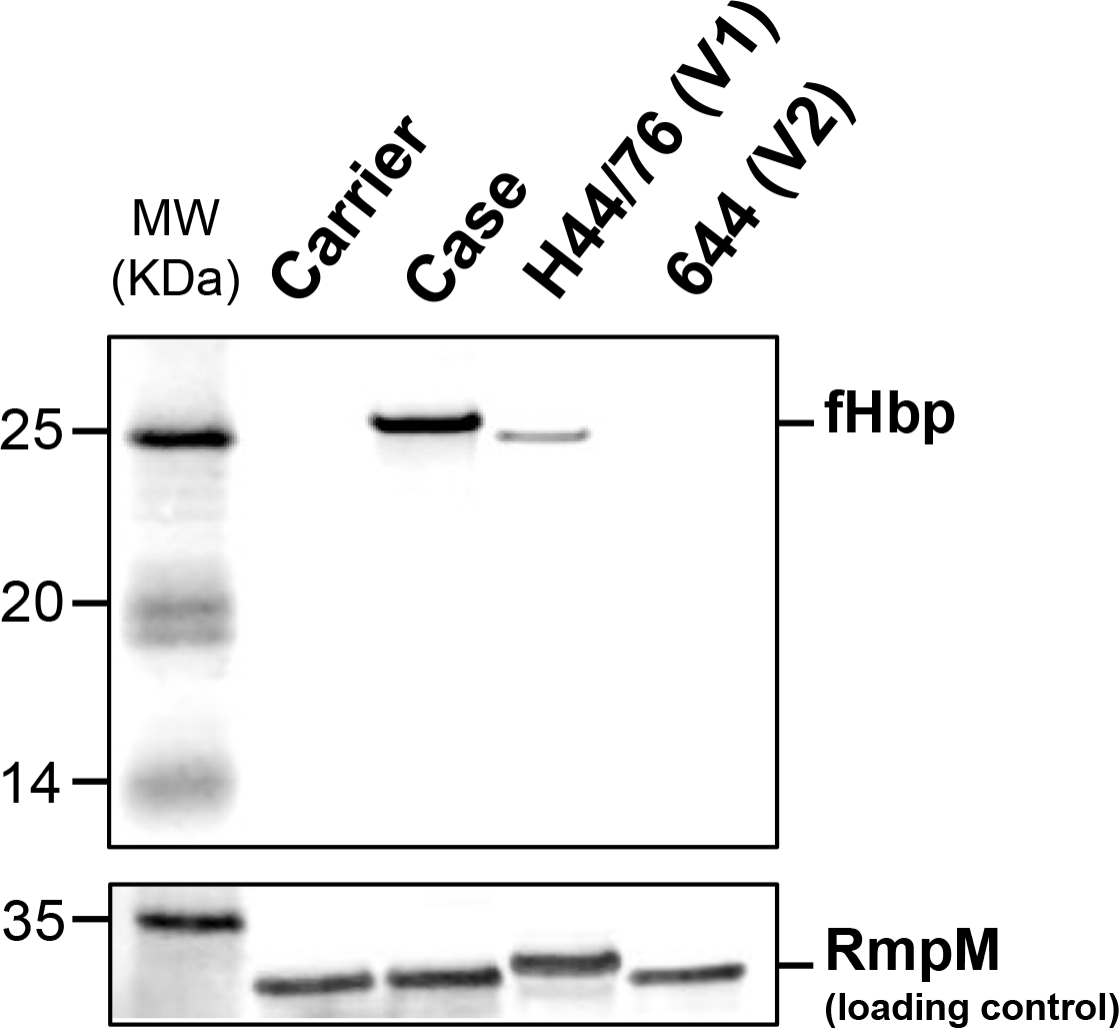
Western blot using anti-fHbp mAb JAR5 (NIBSC 13/216) confirmed fHbp expression in the Case and absence of expression in the Carrier isolate. MenB isolate H44/76 was used as a positive control as it expresses fHbp variant 1 (**v1**), while PubMLST isolate 644 was used as a negative control for v1 as it expresses fHbp variant 2 (**v2**). RmpM polyclonal mouse serum (in house) was used as an internal loading control.

Expression of fHbp in the Case isolate and absence of expression in the Carrier was confirmed by western blot (Fig. 1) and whole-cell ELISA (data not shown) using anti-fHbp mAb JAR5. The JAR5 binding site is located at amino acid position 121, a site predicted to be present in the Carrier if a truncated fHbp is expressed [28].

### fHbp expression accounts for increased serum survival and complement resistance of the Case isolate

fHbp is a key meningococcal virulence factor and plays an important role in the survival of the meningococcus in human serum and resistance to complement killing [29, 30]. To test the impact of fHbp expression in the Case and Carrier isolates on their relative ability to survive in human serum, fHbp expression was complemented in the Carrier isolate using a pGCC4 expression plasmid. The resulting isolate is referred to as ‘erm^R^-fHbp-Carrier’. Expression of fHbp in erm^R^-fHbp-Carrier was induced by overnight incubation in MH broth supplemented with 1 mM IPTG and confirmed via whole-cell ELISA using anti-fHbp mAb JAR5 (Fig. S2). As a transformation control, the Carrier was transformed with pGCC4 plasmid lacking the *fHbp* gene and referred to here as ‘erm^R^-Carrier’.

**Fig. 2.**
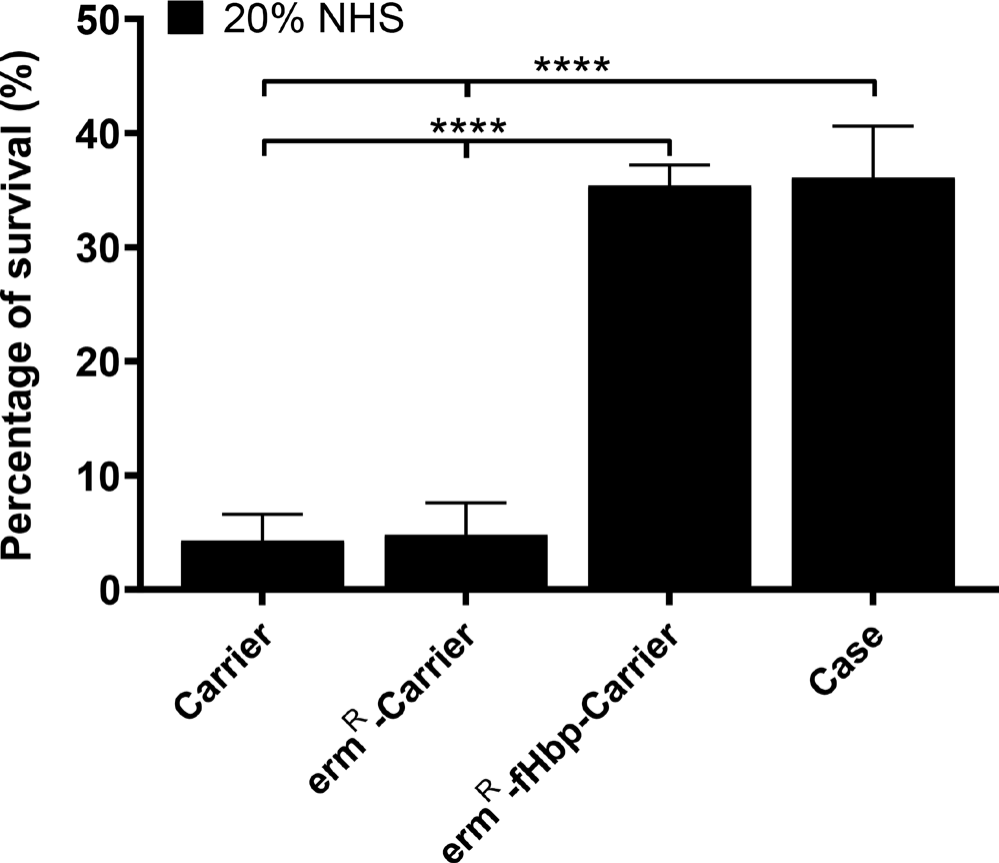
Percentage of survival in 20 % NHS for the Carrier, Case, erm^R^-Carrier and erm^R^-fHbp-Carrier isolates was evaluated using a serum sensitivity assay. Complementation of fHbp expression in the Carrier isolate (erm^R^-fHbp-Carrier isolate) increases this isolate's ability to survive in human serum by ~31 %. Error bars indicate ±sd from three independent experiments. ****P<0.0001.

The Carrier, Case, erm^R^-Carrier and erm^R^-fHbp-Carrier were incubated respectively with 20 % NHS or 20 % HI-NHS as a survival control, where complement components in the serum were destroyed by heating. Following incubation for 1 h, the percentage of survival of each isolate was calculated by comparing the number of colony-forming units recovered from incubation in 20 % NHS with those of the same isolate recovered from incubation in 20 % HI-NHS control. Recovery of 100 % was observed in 20 % HI-NHS control for all isolates. A reduction in serum survival was seen for all isolates in 20 % NHS compared to 20 % HI-NHS. Survival in 20 % NHS serum, however, was significantly increased for both the erm^R^-fHbp-Carrier and the Case isolates (35.4±1.8 and 36.1±4.5 % respectively, mean±sd) compared to both the erm^R^-Carrier and the Carrier isolates (4.8±2.8 and 4.3±2.3 % respectively; Fig. 2), suggesting the importance of fHbp expression in the survival of the meningococcus in human serum and resistance to complement.

### Differential killing of the Case and Carrier with sera containing anti-fHbp antibodies

Susceptibility of the Case and Carrier isolates to sera containing anti-fHbp antibodies may vary due to the highlighted differences in their fHbp expression. Here polyclonal anti-fHbp sera from mice immunized with Bexsero vaccine (GlaxoSmithKline) were used as a source of anti-fHbp antibodies raised against fHbp variant 1.

**Fig. 3.**
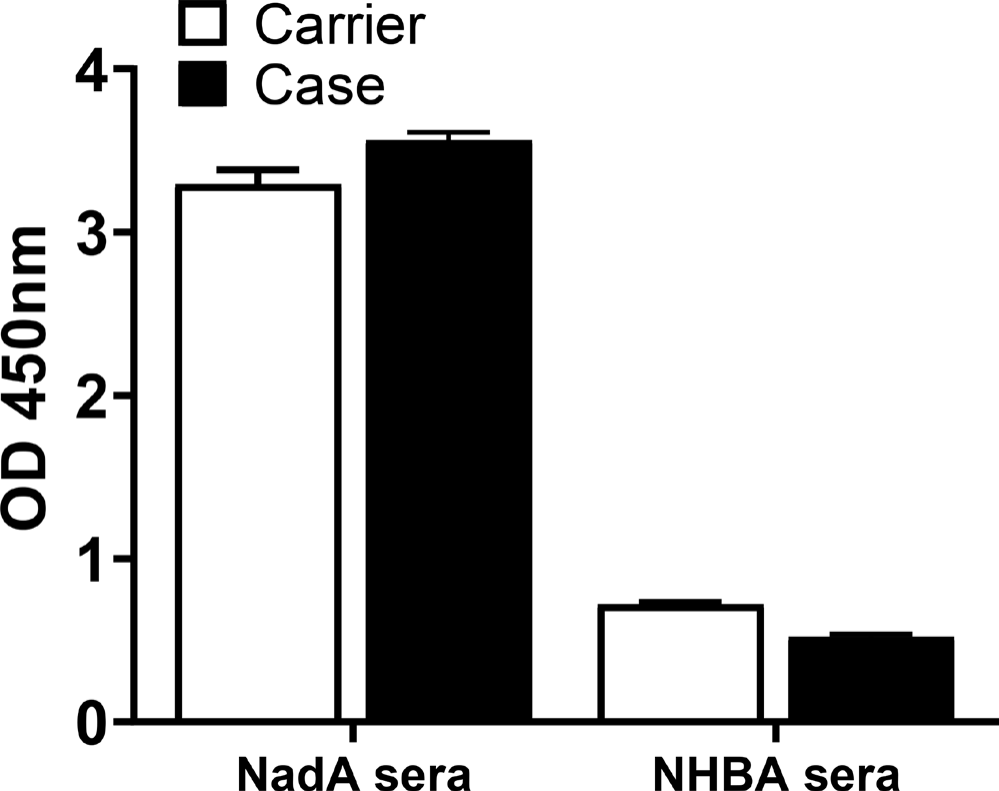
Whole-cell ELISA using anti-NadA and anti-NHBA mouse sera, kindly donated by Dr B. Bolgiano (NIBSC), was used to evaluate reactivity of Case and Carrier isolates to NadA and NHBA antigens respectively. Average results using negative control mouse sera from mice immunized with normal saline, kindly provided by Dr H. Chan (NIBSC), were subtracted from the results.

Bexsero sera also contain antibodies against the other vaccine components, NadA, NHBA and OMVs from the NZ98/254 strain, predominantly to the porin protein PorA [25]. Peptide sequences from the Carrier and Case isolates demonstrated that the isolates belong to the same variant group for NadA and NHBA, but different from Bexsero (Table 2). In addition, as previously shown, both isolates do not express PorA [14]. Whole-cell ELISA using anti-NadA and anti-NHBA mouse sera (Fig. 3) showed comparable responses for both the Carrier and Case isolates, with a strong reactivity to anti-NadA antibodies and a weak reactivity to anti-NHBA antibodies, suggesting that any difference between isolates in their reactivity when using Bexsero mouse sera can be ultimately attributed to fHbp.

**Table 2.** Peptides and variants for each of the four antigens present in Bexsero compared to those in the Carrier and Case isolates

Antigen	Bexsero	Carrier	Case
fHbp	Peptide 1, variant 1	Not expressed∗	Peptide 1, variant 1
NadA	Peptide 8, variant 2/3	Peptide 2, variant 2/3	Peptide 2, variant 2/3
NHBA	Peptide 2	Peptide 20	Peptide 20
PorA	P1.4	Not expressed†	Not expressed†

*As demonstrated in this study, fHbp is not expressed by the Carrier isolate.

†As previously demonstrated elsewhere, PorA is not expressed by both Carrier and Case isolates [14].

The serum bactericidal antibody (SBA) assay was then used to investigate the bactericidal activity of Bexsero sera on the Case and Carrier isolates. The Case had a significantly higher SBA titre (*P*=0.0034) compared with the Carrier (2048 vs. 512, respectively), suggesting that increased killing in the Case isolate is due to anti-fHbp antibodies in the sera binding to fHbp.

### Identification of recombination hotspots provides evidence of recombination

**Fig. 4.**
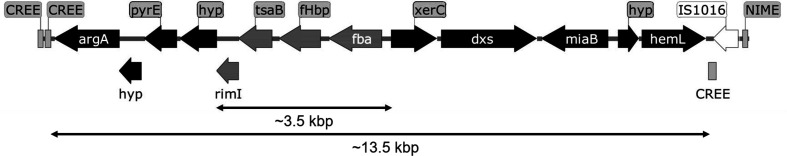
*
Neisseria* Correia repeat enclosed elements (CREE) [31], insertion sequence IS1016 and *Neisseria* intergenic mobile element (NIME) [54] were identified on each side of an ~13.5 kbp region which might have been acquired through horizontal transfer followed by recombination.

Comparison of the Case and Carrier genomes revealed >99 % identity, with 163 nucleotide polymorphisms identified all located within eight genes [15]. Four of these genes, *rimI* (NEIS0347), *tsaB* (NEIS0348), *fHbp* (NEIS0349) and *fba* (NEIS0350)*,* are in an area spanning ~3.5 kbp (Fig. 4), where 142 out of the 163 polymorphisms identified are present. The relatively high number of polymorphisms in this area as compared to the rest of the genome could be explained by a recombination event [15].

To determine whether this ~3.5 kbp DNA fragment was acquired through horizontal gene transfer, the nucleotide sequences immediately upstream and downstream of this area were scrutinized for the presence of recombination hotspots, but no *Neisseria* repeated regions, insertion sequences (IS) or mobile genetic elements were identified. The search was extended and *Neisseria* Correia repeat enclosed elements (CREE) [31] were identified on either side of a sequence spanning ~13.5 kbp (13 439 bp; Fig. 4). Insertion sequence IS1016 was found overlapping with the CREE element next to the *hemL* gene. In addition, *Neisseria* intergenic mobile element (NIME) was also detected adjacent to IS1016. These repeat regions are common targets for the insertion of mobile elements in the genome, and thus a fragment of ~13.5 kbp might have been acquired through horizontal transfer.

### The recombinant DNA fragment in the Case isolate is unique

Assuming that the ~13.5 kbp DNA fragment is mobile and that it has been transferred between isolates through horizontal gene transfer, blast searches were carried out against all isolates available on the National Center for Biotechnology Information (NCBI) database (https://blast.ncbi.nlm.nih.gov/Blast.cgi) to identify the prevalence and possibly the origin of this fragment. The search returned similarities only with *N. meningitidis* isolates, so a more in-depth blast search was then performed against the totality of *Neisseria* isolates available in the PubMLST database (search based on 68 805 isolates available in PubMLST on 29 July 2020).

Nucleotide alignments with the ~13.5 kbp DNA fragment from the Carrier isolate showed that 363 *N *. *meningitidis* isolates have ≥99 % identity to the Carrier in this region, with 68 isolates being 100 % identical, all carrying the ΔT366 frameshift mutation in *fHbp*. These isolates were collected in a timeframe spanning from 1993 to 2019 and included invasive (*n*=37), carrier (*n*=11) or isolates with an unknown epidemiology (*n*=20). All isolates belong to the ST-11 clonal complex, with the majority belonging to serogroup C (*n*=50), while serogroup B (*n*=9) and non-groupable/unclassified isolates (*n*=4/*n*=5 respectively) were also present.

Similar blast nucleotide alignment was applied for the ~13.5 kbp fragment from the Case isolate, with 10 isolates identified showing ≥99 % identity to the Case in this region. All isolates are invasive, belonging to the ST-11 clonal complex, serogroup B (*n*=6) and serogroup C (*n*=4) and none carried the ΔT366 frameshift mutation in *fHbp*. There were no isolates in the PubMLST database with 100 % sequence identity to the ~13.5 kbp Case region and a minimum of 90 SNPs differences were identified, indicating that the acquired fragment in the Case isolate possibly came from a previously unidentified isolate.

## Discussion

Investigation into the closely related Case and Carrier isolates was prompted by their contrasting clinical association, observed during the 1997 IMD outbreak at the University of Southampton, in which one person died and a close contact survived. Previous studies comparing their genomes, MLST, fine typing PFGE and serology profiles indicated that they were indistinguishable, with the exception of differences in eight genes. The aim of this study was to decipher which differences might have affected the virulence of these isolates and influence their ability to cause (or not cause) disease.

Comparative proteomics identified six proteins differentially expressed between the isolates, with four (PrpC, Imp, Fba, AldA) upregulated in the Case isolate, one (PilC2) downregulated and fHbp only expressed by the Case isolate; however, the increased expression of the four upregulated proteins (PrpC, Imp, Fba, AldA) was relatively modest compared to the differential expression of fHbp and PilC2.

Of the four upregulated proteins, fructose-1,6-bisphosphate aldolase (Fba) is noteworthy, as this protein was also previously identified during the WGS comparison [15]. Known for its cytoplasmic role in glycolysis, Fba has additional ‘moonlighting’ functions [32], being a virulence factor with roles in adhesion [33, 34], transcription regulation, host redox homeostasis and immune responses [35] in various organisms. In *N. meningitidis *, it is surface expressed and binds human plasminogen through a lysin (K354) at its C terminus [32, 36]. Thirty-eight nucleotide polymorphisms were identified between the Carrier and the Case *fba*. While most polymorphisms were synonymous, three were non-synonymous, causing amino acid substitutions from carrier to case: V163A, S342N and C343R. The impact of these changes remains unknown, but they could have contributed to the increased virulence of the Case isolate.

PilC2 was the only protein downregulated in the Case isolate. Alongside PilC1, both proteins are responsible for bacterial type four pili (T4P)-mediated adhesion of meningococci to human cells, while promoting different effects on infected cells, perhaps by allowing the bacteria to adapt to different environments [37, 38]. In *N. meningitidis *, only PilC1 is required for adhesion and PilC2, expressed independently of PilC1, fails to promote adhesion despite identical functions in pilus expression and transformation competence [39]. Both proteins are, however, phase variable and present a repeated guanine (G)-tract that is responsible for their phase variable status [40]. One synonymous substitution (G→C) in the *PilC2* poly-G tract has been seen here between the Carrier and the Case, with no changes in protein sequence or its on/off status. Downregulation of this protein in the Case isolate might have occurred as pili expression is no longer required when the bacterium is located in the CSF [41, 42].

The most notable result from the comparative proteomic analysis is the absence of peptides detected for fHbp in the Carrier isolate. Lack of fHbp expression in the Carrier is caused by a single base deletion (ΔT366) in *fHbp*, while a full-length protein is expressed by the Case. Isolates lacking fHbp can retain their ability to cause disease [27] through expression of alternative receptors which bind factor H (fH) [43], such as NspA [44] and PorB2/3 alleles [45]. However, no differences in expression levels or encoding genomic sequence were identified for these alternative receptors.

The Case isolate showed an enhanced survival rate in human serum as compared to the Carrier isolate. Furthermore, when fHbp was complemented into the Carrier isolate its survival in serum was augmented to be comparable with that of the Case. Meningococci commonly reside in the oropharynx without causing disease, but can occasionally enter the bloodstream, where they face a hostile environment. In response, they have evolved the ability to evade human immune responses [46] and to survive complement-mediated killing. One such mechanism is the expression of fHbp [30, 47, 48], an inhibitor of the alternative complement pathway [49, 50]. Binding of fH by the meningococcus protects it from complement-mediated killing via the alternative pathway and shields the organism from the innate immune response [49]. The improved survival in serum conferred by fHbp in the Case isolate could translate into its improved survival in the human host, and thus explain the apparent increase in virulence of the Case versus the Carrier. In addition, whilst not quantitatively confirmed, the Case appears to express a high amount of fHbp (as shown via Western blot in Fig. 1) as compared to H44/76, an isolate known to express high levels of this protein [30]. Analysis of the fHbp upstream region (^igr_up^NEIS0349 [51]) for the Case (allele 21), the Carrier (allele 5) and H44/76 (allele 6) isolates revealed eight SNPs between the Case and the Carrier and five SNPs between the Case and H44/76 (Table S2). Only 10 isolates on PubMLST have been found to present identical ^igr_up^NEIS0349 and *fHbp* alleles to the Case isolate and all isolates were identified from patients with invasive disease. It could be that expressing high levels of fHbp contributes to the ability of the Case isolate to survive in serum and crucially increases the virulence of the isolate. Further work to understand levels of fHbp expression in strains with ^igr_up^NEIS0349 is required.

Evaluation of susceptibility of the Case and Carrier isolates to sera containing anti-fHbp antibodies was performed using polyclonal mice sera from mice immunized with Bexsero (GlaxoSmithKline). Bexsero contains four antigens, PorA (as part of an outer membrane vesicle), fHbp, NadA and NHBA. Due to the presence of antibodies against the homologous NadA and NHBA protein antigens, the sera demonstrated killing activity against both the Case and Carrier isolates. However, an increased killing of the Case isolate compared to the Carrier isolate was observed, which we attribute to the Case isolate expressing the fHbp antigen. In addition to Bexsero, another vaccine approved for prevention of meningococcal group B disease is the bivalent-fHbp vaccine Trumemba (Pfizer) [52]. This vaccine contains two lipidated fHbp variants. In this study, sera from mice immunized with Trumenba were unavailable for comparison to Bexsero. However, as Trumenba contains the homologous variant 1 fHbp antigen as expressed by the Case isolate, it is highly likely that Trumenba would evoke a protective response against the Case isolate similar to Bexsero. In contrast, as Trumenba does not contain any antigens expressed by the Carrier isolate we would not expect the vaccine to evoke a response against this isolate.

The levels of sequence identity between the Carrier and the Case isolates, obtained from oropharynx and CSF respectively, indicated the isolates shared a recent common ancestor. Since the Carrier isolate was retrieved from a person involved in mouth-to-mouth resuscitation of the Case, presumably there was transmission of meningococci from the ‘Case oropharynx’ (oropharynx of the student who died of IMD caused by the Case isolate) to the ‘Carrier oropharynx’ (oropharynx of the survivor, who performed mouth-to-mouth resuscitation of the student who died, without contracting the disease). It has been suggested that a transmission event during mouth-to-mouth resuscitation followed by subsequent homologous recombination might have occurred [15]. In this study we identified the presence of Correia elements [53], IS1016 and NIME flanking an ~13.5 kbp DNA fragment comprising the four genes (*rimI*, *tsaB*, *fHbp*, *fba*) where the majority of nucleotide changes were identified [15].

The presence of these genetic elements could facilitate DNA exchange between *Neisseria* co-residing in the oropharynx. To understand the origin of this ~13.5 kbp recombinant fragment, blast of the Carrier ~13.5 kbp region against *Neisseria* isolates in the PubMLST.org/neisseria database identified 68 meningococcal genomes sharing 100 % nucleotide similarity in this region. In contrast, no meningococci with 100 % similarity to the Case ~13.5 kbp region were detected. This suggests that only the Carrier recombinant fragment has continued to circulate in the population in contrast to the unique Case recombinant fragment. Death of the human host is ultimately lethal also for the meningococcus and, as the Case ~13.5 kbp fragment does not seem to have persisted in the meningococcal population, it may represent an evolutionary dead end.

Sequence data in themselves do not necessarily provide evidence of direct transmission between Case and Carrier, although in this case the epidemiological data were compelling. In addition, sequence homology does not establish the origin of the recombinant fragment identified. blast analysis against other species and genera did not identify any sequence matches in databases at the time of analysis. Despite the very close relationship of these identical isolates, a significant difference in their ability to survive in serum was demonstrated and it was established that this differential survival rate was due the expression of fHbp in the Case isolate, which enhanced its resistance to complement-mediated killing compared to the Carrier isolate, which lacked fHbp expression. Susceptibility of the Case isolate to mice anti-sera raised against an fHbp-containing vaccine, Bexsero, also reinforced the role of fHbp in virulence and immunity. The enhanced virulence of the CSF-isolated meningococcus, Case, was mediated by fHbp and this probably played a major role in causing IMD and ultimately death of a student by the Case isolate.

## Supplementary Data

Supplementary material 1
