# Supplementary material for: Factor H binding protein (fHbp)-mediated differential complement resistance of a serogroup C Neisseria meningitidis isolate from cerebrospinal fluid of a patient with invasive meningococcal disease
Source: Access Microbiol. 2021 Sep 9;3(9):000255. doi: 10.1099/acmi.0.000255 (PMC8549389; doi:10.1099/acmi.0.000255)
Supplement: Supplementary material 1 [file acmi-3-0255-s001.pdf]

Supplementary Figures and Tables

Supplementary Table 1. Primers and probes used in this study.

Primer	Sequence (5' → 3')	Use in this study for:
gdh **forward primer**	GAAGGGCGTTCCCTTCTAC	qRT-PCR
prpC **forward primer**	TTCTTGAGCAACGCAAAGAC	
imp **forward primer**	GCCGCGTTTATCTTATTGGT	
fba **forward primer**	CTTGATCATGCTGCCGAA	
aldA **forward primer**	ACACGCTCGACCAAGTCAT	
gdh **reverse primer**	AAACGATTTCCGCCACTTT	
prpC **reverse primer**	TCTTCAATCTCCACAAACGC	
imp **reverse primer**	ATGGGAATCATCGGAATCAG	
fba **reverse primer**	GCACTCGCCTGTACGATG	
aldA **reverse primer**	GCGGGTAACGTAGAAGGCT	
gdh **probe**	CTGCGTACCGGCAAACGCAT	
prpC **probe**	TCATCCGTCCGAGCGCAAAC	
imp **probe**	AACCTGCCCGGTACGCACG	
fba **probe**	TCGAACAAATGCGCGCCATT	
aldA **probe**	CGCACTGGCTAACGATTGCGA	
**erm^R-Carrier forward primer**	TGGCTGTGCAGGTCGTAAAT	Screening of erm^R-Carrier colonies
**erm^R-Carrier reverse primer**	TCCTGAAGACATCCGCCAAC	
**erm^R-fHbp-Carrier forward primer**	GGCGATTTCAAATGTTCGAT	Screening of erm^R-fHbp-Carrier colonies
**erm^R-fHbp-Carrier reverse primer**	TGGCTGTGCAGGTCGTAAAT	

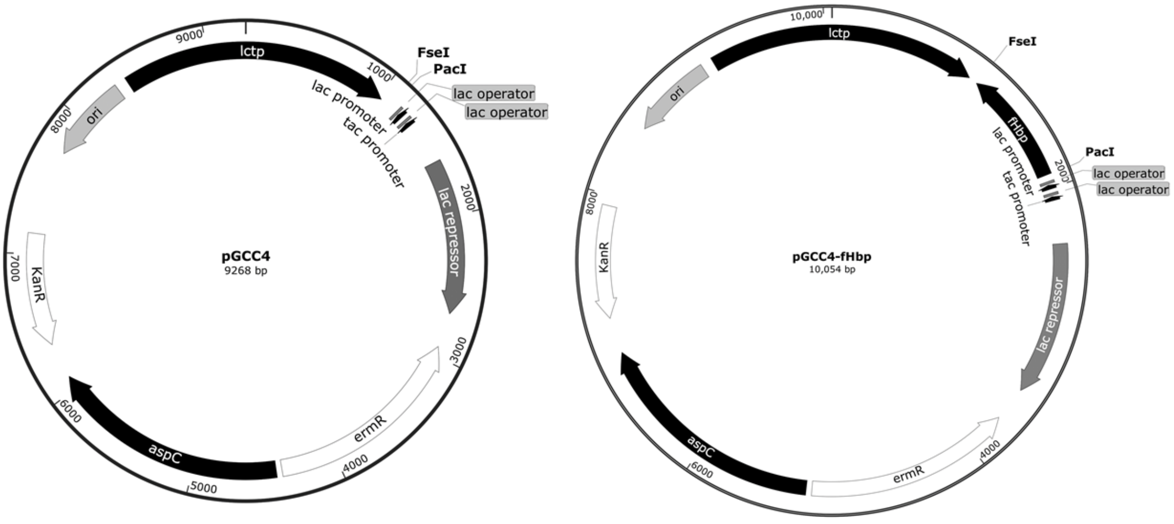

***Supplementary Figure 1*.** Schematic representations made using SnapGene (version 4.3.10.0; GSL Biotech LLC) of pGCC4 constructs, kindly provided by Prof. C.M. Tang (Sir William Dunn School of Pathology, University of Oxford). Factor H binding protein (fHbp V1 from MC58) was cloned at *PacI* and *FseI* restriction sites between *lctP* and *aspC* loci in pGCC4 to obtain pGCC4-fHbp plasmid construct. PGCC4 alone or containing fHbp were subsequently incubated with the Carrier isolate to allow transformation and recombination to obtain erm^{R}-Carrier and erm^{R}-fHbp-Carrier isolates respectively. FHbp expression in erm^{R}-fHbp-Carrier isolate was later achieved by overnight incubation in MH broth supplemented with 1 mM IPTG (Thermo Fisher Scientific).

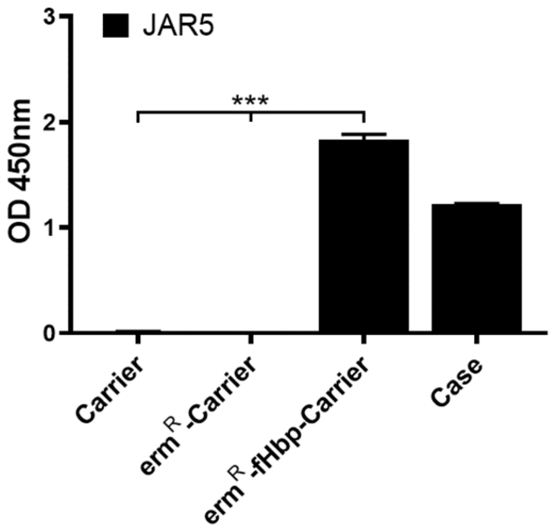

Supplementary Figure 2. Whole-cell ELISA was performed on the Carrier, Case, fHbp complemented (erm^{R}-fHbp-) and non-complemented (erm^{R}-) Carrier using anti-fHbp mAb JAR5. FHbp was detected only in the Case and erm^{R}-fHbp-Carrier isolates (after overnight incubation in 1 mM IPTG to allow fHbp expression).

Supplementary Table 2. Summary of the SNPs identified in the upstream intergenic region of fHbp (^{igr_up}NEIS0349) between the Carrier (allele 5), Case (allele 21) and H44/76 (allele 6) isolates.

**^{igr_up}NEIS0349 Nucleotide changes**			
**Position** (bp)	**Carrier** (allele 5)	**Case** (allele 21)	**H44/76** (allele 6)
8	A	C	C
35	C	T	C
36	C	T	C
46	G	G	A
47	C	T	C
76	C	T	C
90	A	G	G
113	G	A	A
120	T	A	A
